# 

**Published:** 1906-01-15

**Authors:** 


### CONTENTS.

Event and Comment - - - - - - - - - - 1

Business in Dental Practice, - - By George F. Burke, D.D.S. 6

Avoiding Accidents and Failures in Anesthesia,

By Egbert T Loeffler, B.S., D.D.S. 17

Funomia, - - - - - - By Toothsom Topics Tuller. 21

The Chemistry of Plaster of Paris,

By William J. Manning, M.D., D.D.S. 25

Pyorrhea Treatment, A New Feature, - - By [illegible] Ames 30

Bibliography - - - - - - - - - - - 34

Obituary, - - - - - - - - - - - - 36

Indents - - - - - - - - - - - - 39

Announcements - - - - - - - - - - - 48

